# Clinical impact of *EZH2* and its antagonist *SMARCA4* in ovarian cancer

**DOI:** 10.1038/s41598-020-77532-x

**Published:** 2020-11-23

**Authors:** Katharina Leitner, Irina Tsibulak, Verena Wieser, Katharina Knoll, Daniel Reimer, Christian Marth, Heidi Fiegl, Alain G. Zeimet

**Affiliations:** Department of Obstetrics and Gynecology, Innsbruck Medical University, Anichstraße 35, 6020 Innsbruck, Austria

**Keywords:** Chromatin remodelling, Ovarian cancer, Ovarian cancer

## Abstract

SMARCA4 and EZH2 are two functional key players of their respective antagonizing chromatin remodeling complexes SWI/SNF and PRC2. EZH2 inhibitory drugs may abrogate pro-oncogenic features of PRC2 and turn the balance to cell differentiation via SWI/SNF activity in cancers. *SMARCA4* and *EZH2* expression was assessed by RT-PCR in 238 epithelial ovarian cancers (OCs) and put in relation to clinico-pathological parameters and patients’ outcome. Optimal thresholds for high and low expression of both variables were calculated by the Youden’s index based on receiver operating characteristic (ROC) curves. High *SMARCA4* mRNA expression was independently associated with favorable progression-free survival (PFS) (*P* = 0.03) and overall survival (OS) (*P* = 0.018). As Youden’s threshold determination for *EZH2* yielded a S-shaped ROC-curve, two cut-off points (29th and 94th percentile) predicting opposite features were defined. Whereas *EZH2* mRNA levels beyond the 29th percentile independently predicted poor PFS (*P* = 0.034), Cox-regression in *EZH2* transcripts above the 94th percentile revealed a conversion from unfavorable to favorable PFS and OS (*P* = 0.009 and *P* = 0.032, respectively). High *SMARCA4* expression associates with improved survival, whereas moderate/high *EZH2* expression predicts poor outcome, which converts to favorable survival in ultra-high expressing OCs. This small OC subgroup could be characterized by REV7-abrogated platinum hypersensitivity but concomitant PARP-inhibitor resistance.

## Introduction

Ovarian cancer (OC) is the leading cause of death in women with gynecological malignancies in the developed world^1, 2^. Although during the last decade implementation of a number of new treatment approaches against OC led to an improvement of prognosis, the latter still remains disastrous^3^.

The accessibility of DNA to regulatory transcription machinery proteins is regulated by antagonizing classes of enzymes which are found in two large ATP-dependent protein complexes, the so-called chromatin remodeling complexes, namely the switch/sucrose non-fermentable (SWI/SNF) complex and the covalent histone-modifying complexes, like the polycomb repressive complex (PRC2). These complexes regulate the structure of chromatin by either activating or repressing functions and thereby are crucial for genomic transcription and gene expression^4,5^.

The SWI/SNF complex uses the energy from ATP hydrolysis to weaken interactions between DNA and histones and thereby regulates the access to genes for the mechanisms of DNA transcription, replication and repair^6,7^. Members of the SWI/SNF complex are recurrently mutated and inactivated in about 20% of human cancers^8^ and the tumor suppressive function of SWI/SNF is well documented. The human SWI/SNF complex contains only one of the two mutually exclusive catalytic subunits with ATPase activity—either SMARCA2 (BRM) or SMARCA4 (BRG1)—and several other subunits, including ARID1A and ARID1B, which are mutated in a variety of cancers^6,7^. Mutations of ARID1A can be found in ovarian clear-cell and endometrioid carcinomas^9^ as well as in high-grade endometrial carcinomas^10^. *ARID1B* can be used as a prognostic and predictive biomarker in breast cancer^11^. PBRM1, another gene of SWI/SNF complex is frequently mutated in renal cancer^12^. Malignant rhabdoid tumors often harbor mutations of SMARCB1^13^.

The expression of *SMARCA4*, which is one of the main topics in this paper, is absent in around 10% of human primary non-small-cell lung cancers (NSCLC) and its loss is associated with poor patient survival^14^. Interestingly, inactivating biallelic mutations of *SMARCA4* gene can be found in nearly all small cell carcinoma of the ovary of the hypercalcemic type (SCCOHT), which was first described in 1979 by R. Scully^15–18.^ SCCOHT is a very rare but aggressive form of ovarian cancer diagnosed especially in young women under the age of 40 years. It is characterized by elevated paraneoplastic serum calcium levels and often occurs within families^15,19^. Recurrence is rapid and approximately two thirds of patients with advanced disease die within 2 years after diagnosis^20^.

In contrary, proteins of the PRC2 complex play an important role in oncogenic transformation^21^. Its catalytic subunit EZH2 (Enhancer of zeste homolog 2) trimethylates lysine 27 of histone H3 (H3K27me) and thereby promotes transcriptional silencing. *EZH2* is highly expressed in various tumors, such as breast cancer, prostate cancer, endometrial cancer and melanoma, where it often correlates with advanced tumor stages and poor prognosis^22–24^. Recently *E2F1* was found to directly regulate transcription of *EZH2* and *SUZ12*, another component of the PRC2 complex. Furthermore, *E2F1-EZH2-SUZ12* signature predicts aggressiveness and impaired prognosis in bladder cancer^25^. In epithelial OCs *EZH2* is also often overexpressed and thereby promotes proliferation and invasion of tumor cells^26^.

Inhibition of EZH2 leads to reduced tumor formation and growth. Therefore, EZH2 inhibitors, such as tazemetostat, could play a potential role in the treatment of many cancers^27,28^. The Food and Drug Administration granted recently accelerated approval to tazemetostat for metastatic or locally advanced epithelioid sarcoma not eligible for complete resection.

In SCCOHT it was shown that EZH2 inhibitors induced cell cycle arrest, apoptosis and cell differentiation in tumor cells as well as suppression of tumor growth and improved survival in murine models^18^. Furthermore, in high-grade serous cancers (HGSOC) another mechanism involving *EZH2* was recently reported in CARM1 overexpressing cancers. Herein authors showed that EZH2 inhibitors sensitizes this OC subtype to PARP inhibitors despite its proficiency in homologous recombination DNA repair^29^.

The high prevalence of SWI/SNF mutations in various human malignancies, the occurrence of *SMARCA4* as the driver mutation in SCCOHT and the potential therapeutic strategy of EZH2 inhibitors led us to explore the role of *SMARCA4* and *EZH2* in epithelial ovarian cancers. Therefore, we determined *SMARCA4* and *EZH2* expression on the transcriptome level in 238 fresh-frozen epithelial OCs and evaluated a putative biological role of both representatives of the two antagonistic chromatin remodeling complexes by analyzing their associations with clinico-pathological characteristics and using data on patients’ survival as benchmarks.

## Results

**Figure 1:**
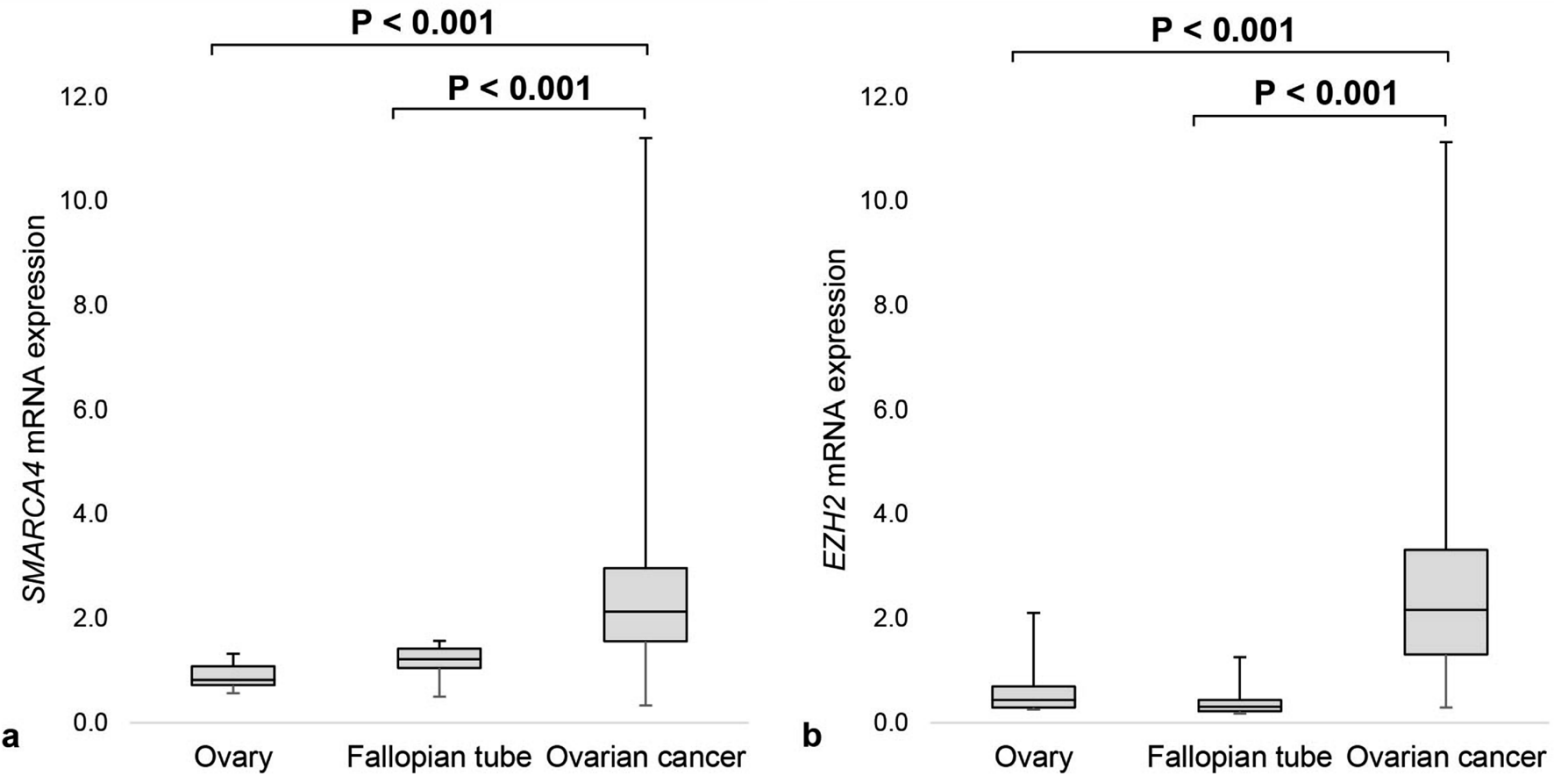
*SMARCA4* and *EZH2* mRNA expression in ovarian tissues. (**a**) *SMARCA4* mRNA expression in 16 non-neoplastic ovaries, 19 non-neoplastic fallopian tubes and 238 OC tissues, (**b**) *EZH2* mRNA expression in 16 non-neoplastic ovaries, 19 non-neoplastic fallopian tubes and 238 OC tissues.

We analyzed mRNA expression of *SMARCA4* and *EZH2* in 238 OC, nineteen samples of non-neoplastic and otherwise histologically normal fallopian tube mucosa and sixteen histologically normal ovarian tissues. Median *SMARCA4* and *EZH2* mRNA levels in OC tissues compared to normal fallopian tubes were 1.7-fold and 7.2-fold higher, respectively (*P* < 0.001 for both) and 2.6-fold and 5.1-fold higher compared to non-neoplastic ovarian tissues (*P* < 0.001 for both) (Fig. 1).

While no relevant differences in *SMARCA4* expression between the various histological OC subtypes were found, significant differences between histological subtypes were pointed out in *EZH2* mRNA expression (*P* < 0.001). In detail, highest levels of *EZH2* expression were found in the pooled group of high-grade serous (HGSOC) and endometrioid (HGEOC) cancers (*P* < 0.001). However, it is noteworthy, that in endometrioid cancers *EZH2* mRNA levels were essentially the same regardless whether cancers were assigned to high-grade or low-grade cancers. This was not the case in cancers with serous histology, where significantly lower *EZH2* levels were found in low-grade serous (LGSOC), as compared with high-grade serous OCs (*P* < 0.001); (Table 1).

**Table 1:** Association of *SMARCA4* and *EZH2* mRNA expression with clinico-pathological characteristics in 238 OC patients.

Variable	Number (%)	mRNA expression values (arbitrary units)			
		*SMARCA4*		*EZH2*	
		Median (IQR)	*P* value	Median (IQR)	*P* value
**Age**					
≤ 62 years (median age)	120 (50.4%)	2.250 (1.74–3.35)	0.064	2.135 (1.24–3.90)	0.487
> 62 years	118 (49.6%)	2.056 (1.46–2.85)		2.185 (1.32–3.03)	
≤ 51.5 years	53 (22.3%)	2.187 (1.63–3.54)	0.381	1.660 (0.75–3.91)	0.060
> 51.5 years	185 (77.7%)	2.099 (1.56–2.87)		2.300 (1.49–3.24)	
≤ 70 years	167 (70.2%)	2.219 (1.71–3.20)	**0.028**	2.270 (1.41–3.73)	0.168
> 70 years	71 (29.8%)	2.005 (1.32–2.81)		1.940 (1.25–2.94)	
≤ 40 years	14 (5.9%)	1.961 (1.13–2.46)	0.151	1.155 (0.66–1.90)	**0.004**
> 40 years	224 (94.1%)	2.163 (1.61–3.04)		2.255 (1.47–3.36)	
**Histology**					
HGSOC	144 (60.5%)	2.250 (1.72–3.07)	0.695	2.390 (1.51–3.69)	** < 0.001**
LGSOC	12 (5.0%)	1.876 (1.53–2.48)		0.710 (0.56–1.52)	
HGEOC	31 (13.0%)	2.392 (1.15–3.98)		2.530 (1.78–5.39)	
LGEOC	13 (5.5%)	2.093 (1.76–2.55)		2.520 (1.59–3.16)	
Clear cell OC	11 (4.6%)	2.216 (1.13–3.33)		1.310 (0.86–2.88)	
Mucinous OC	27 (11.3%)	2.123 (1.44–3.08)		1.570 (0.80–2.56)	
**FIGO stage**					
I/II	64 (26.9%)	2.313 (1.58–3.35)	0.193	2.125 (1.27–3.10)	0.659
III/IV	174 (73.1%)	2.082 (1.57–2.89)		2.185 (1.39–3.41)	
**Tumor grade**					
Low grade	30 (12.6%)	1.915 (1.55–2.47)	0.145	1.410 (0.68–2.54)	**0.001**
Grade III	208 (87.4%)	2.219 (1.58–3.09)		2.320 (1.48–3.58)	
**Residual disease**					
Complete debulking	114 (47.9%)	2.219 (1.69–3.37)	**0.044**	2.125 (1.19–3.29)	0.647
Any residual	114 (47.9%)	2.076 (1.48–2.76)		2.185 (1.48–3.33)	
Not indicated	10 (4.2%)	–		–	
**Subgroups**					
“Low-grade OC”	25 (11.8%)	1.917 (1.61–2.50)	0.188	1.590 (0.71–2.61)	**0.006**
“High-grade OC”	186 (88.2%)	2.250 (1.65–3.08)		2.410 (1.52–3.87)	
**BRCA1 mutation status**					
Wild-type	155 (65.1%)	2.093 (1.64–2.87)	0.587	2.060 (1.25–3.22)	**0.013**
*BRCA1* mutated	35 (14.7%)	2.437 (1.79–3.20)		3.070 (1.90–4.51)	
Not indicated	48 (20.2%)	–		–	
**BRCA2 mutation status**					
Wild-type	181 (76.0%)	2.111 (1.65–2.89)	0.787	2.240 (1.41–3.29)	0.313
*BRCA2* mutated	9 (3.8%)	1.904 (1.55–3.37)		3.910 (1.10–7.05)	
Not indicated	48 (20.2%)	–		–	

*IQR* interquartile range.

Bold values indicate *P* < 0.05.

### *SMARCA4* and *EZH2* transcript levels related to clinico-pathological characteristics of OC patients

Associations of *SMARCA4* and *EZH2* expression with various clinico-pathological parameters are summarized in Table 1. Subgroup analyses are shown in Supplementary Table S1 and S2.

No association with age at time of diagnosis was revealed for either *SMARCA4* or *EZH2* expression when median age in the collective (62 years) or the median age at menopause in Europe (51.5 years)^30^ was used as a cut-off. Nonetheless, we have been interested whether the expression of both antagonistic variables is equally distributed independently of age or linked to specific age groups. We revealed significantly lower *SMARCA4* mRNA expression in patients older than 70 years at the time of diagnosis (*P* = 0.028) and in contrast, lower *EZH2* expression in young patients under 40 years of age (*P* = 0.004).

In “high-grade OCs” elevated *SMARCA4* transcripts were associated with complete resection after surgical debulking (*P* = 0.016) (Supplementary Table S1).

In contrast, *EZH2* expression was higher in the subgroup of “high-grade OCs” (*P* = 0.006). Furthermore, there was a significantly higher *EZH2* mRNA expression in cancers with confirmed *BRCA1* mutations (*P* = 0.013). However, no significant difference in *EZH2* transcript levels was related to the *BRCA2* mutational status**,** but it is worth to note that the number of included cases with *BRCA2* mutations was very low (n = 9).

In the whole OC cohort, a strong positive correlation between *SMARCA4* and *EZH2* expression was uncovered (r^s^ = 0.392, *P* < 0.001). Furthermore, both were directly associated with *BRCA1* and *BRCA2* mRNA expression and with cell cycle promoting regulator *E2F3a*, whereas *EZH2* was additionally associated with the cell cycle promotor *E2F1* (Supplementary Table S3).

### Survival analysis related to the expression of ***SMARCA4*** and ***EZH2***

**Figure 2:**
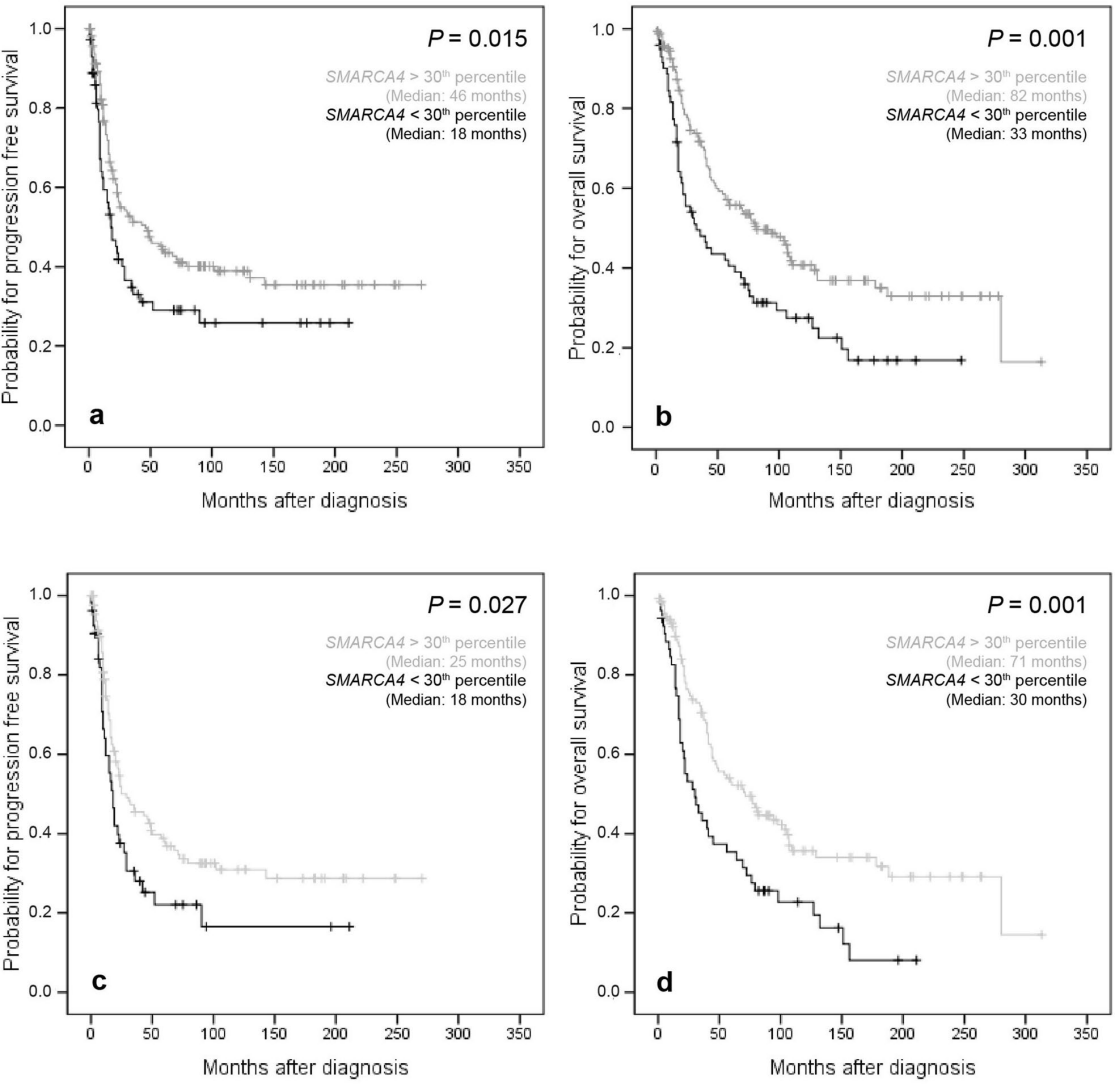
Kaplan–Meier survival analysis and *SMARCA4* mRNA expression according to the 30th percentile as cut off-value in the whole cohort and in the subgroup of “high-grade OC” patients. (**a**) Progression free survival in 238 OC patients, (**b**) overall survival in 238 OC patients, (**c**) progression free survival in 186 “high-grade OC” patients, (**d**) overall survival in 186 “high-grade OC” patients.

Univariate survival analysis in the entire cohort of OC patients showed that high *SMARCA4* mRNA expression was associated with a favorable PFS (*P* = 0.015) and OS (*P* = 0.001). The same was especially true for the subgroup of “high-grade OCs” (*P* = 0.027 and *P* = 0.001, respectively) (Fig. 2). In “low-grade OCs” no clinical prognostic impact could be found for either PFS or OS which could be caused by the low number of included samples (n = 30).

In multivariate Cox-regression analysis *SMARCA4* mRNA expression retained independent prognostic significance for both PFS and OS when the whole collective of OCs was considered (HR 0.66, *P* = 0.03 and HR 0.65, *P* = 0.018, respectively) (Table 2). However, in the subgroup of “high-grade OCs” independency of prognostic impact was only disclosed for OS (HR 0.64, *P* = 0.025) (Table 3).

**Table 2:** Multivariate survival analysis in 238 OC patients. Analysis in (A) EZH2 high expressing cancers and (B) EZH2 ultra-high expressing cancers.

Variable		Progression free survival		Overall survival	
		HR (95% CI)	*P* value	HR (95% CI)	*P* value
**A: EZH2 high expressing cancers**					
Age	Low versus high	1.63 (1.11–2.39)	**0.013**	2.49 (1.74–3.56)	** < 0.001**
FIGO stage	I/II versus III/IV	2.74 (1.49–5.03)	**0.001**	1.44 (0.84–2.47)	0.188
Grading	Low grade versus grade III	1.74 (0.76–3.95)	0.189	2.59 (1.09–6.13)	**0.031**
Residual disease	No versus yes	2.2 (1.44–3.35)	** < 0.001**	2.5 (1.63–3.84)	** < 0.001**
*SMARCA4* (30th percentile)	Low versus high	0.66 (0.45–0.96)	**0.03**	0.65 (0.46–0.93)	**0.018**
*EZH2* (29th percentile)	Low versus high	1.62 (1.04–2.52)	**0.034**	1.23 (0.83–1.82)	0.305
**B: EZH2 ultra-high expressing cancers**					
Age	Low versus high	1.54 (1.05–2.25)	**0.027**	2.42 (1.70–3.45)	** < 0.001**
FIGO stage	I/II versus III/IV	2.98 (1.64–5.43)	** < 0.001**	1.57 (0.91–2.69)	0.102
Grading	Low grade versus grade III	2.32 (1.05–5.10)	**0.037**	2.92 (1.25–6.81)	**0.013**
Residual disease	No versus yes	2.14 (1.40–3.27)	** < 0.001**	2.38 (1.55–3.65)	** < 0.001**
*SMARCA4* (30th percentile)	Low versus high	0.81 (0.56–1.17)	0.26	0.73 (0.51–1.04)	0.079
*EZH2* (94th percentile)	Low versus high	0.21 0.07–0.68)	**0.009**	0.4 (0.16–1.01)	**0.032**

*HR* hazard ratio, *CI* confidence interval.

Bold values indicate *P* < 0.05. The significance level was determined by Cox regression analysis.

**Table 3:** Multivariate survival analysis in 186 “high-grade OC” patients. Analysis in (A) EZH2 high expressing cancers and (B) EZH2 ultra-high expressing cancers.

Variable		Progression free survival		Overall survival	
		HR (95% CI)	*P* value	HR (95% CI)	*P* value
**A: EZH2 high expressing cancers**					
Age	Low versus high	1.74 (1.14–2.66)	**0.010**	2.40 (1.61–3.58)	** < 0.001**
FIGO stage	I/II versus III/IV	2.06 (1.03–4.14)	**0.041**	1.15 (0.62–2.13)	0.658
Residual disease	No versus yes	2.51 (1.56–4.06)	** < 0.001**	2.68 (1.64–4.37)	** < 0.001**
*SMARCA4* (30th percentile)	Low versus high	0.76 (0.50–1.15)	0.189	0.64 (0.43–0.95)	**0.025**
*EZH2* (29th percentile)	Low versus high	1.67 (1.01–2.74)	**0.045**	1.45 (0.92–2.28)	0.108
**B: EZH2 ultra-high expressing cancers**					
Age	Low versus high	1.66 (1.09–2.52)	**0.017**	2.28 (1.54–3.37)	** < 0.001**
FIGO stage	I/II versus III/IV	2.50 (1.25–5.00)	**0.010**	1.32 (0.71–2.45)	0.378
Residual disease	No versus yes	2.36 (1.46–3.80)	** < 0.001**	2.46 (1.51–4.01)	** < 0.001**
*SMARCA4* (30th percentile)	Low versus high	0.95 (0.63–1.44)	0.819	0.74 (0.50–1.09)	0.124
*EZH2* (94th percentile)	Low versus high	0.20 (0.06–0.65)	**0.007**	0.43 (0.17–1.07)	0.069

*HR* hazard ratio.

Bold values indicate *P* < 0.05. The significance level was determined by Cox regression analysis.

**Figure 3:**
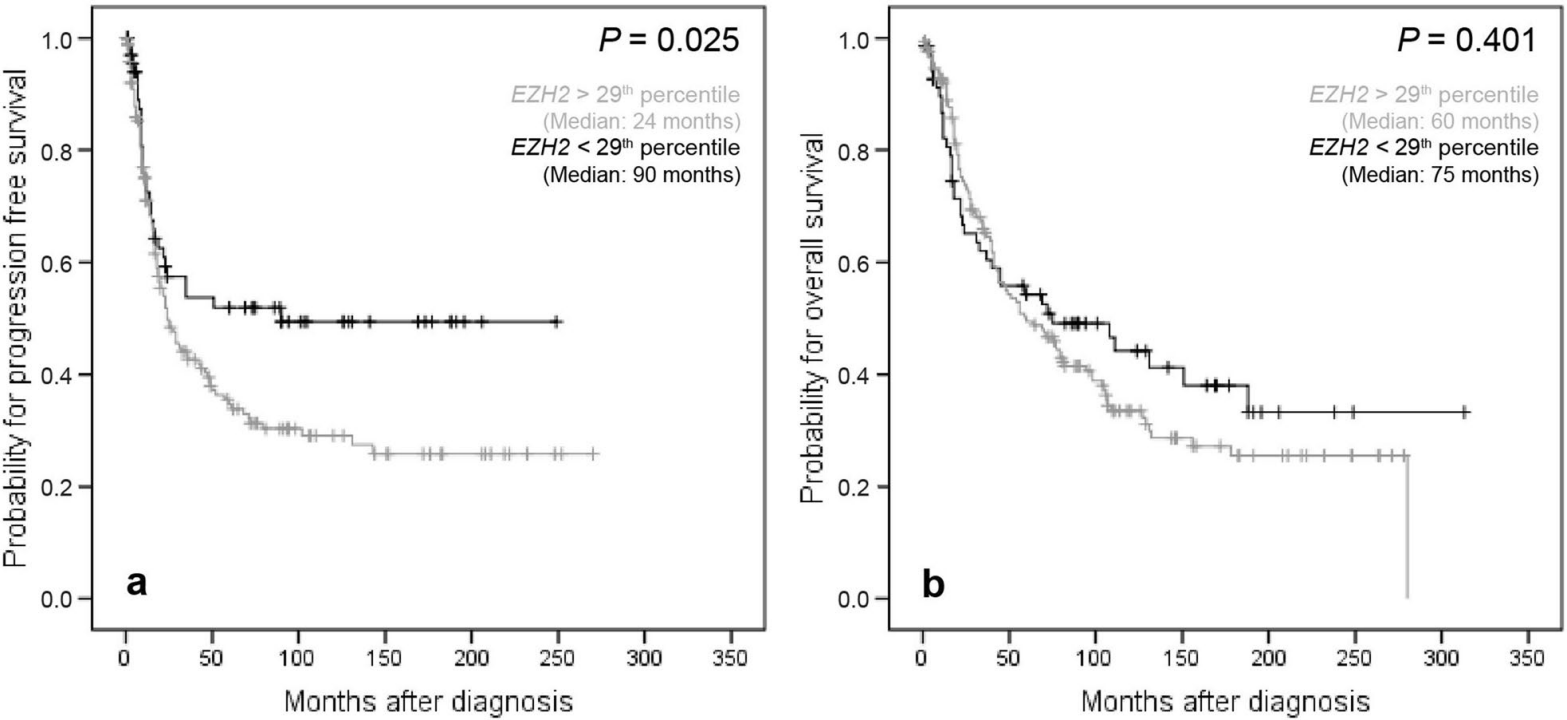
Kaplan–Meier survival analysis and *EZH2* mRNA expression according to the 29th percentile as cut-off value in the whole cohort. (**a**) Progression free survival in 238 OC patients, (**b**) overall survival in 238 OC patients.

As Youden’s threshold determination for *EZH2* yielded a S-shaped ROC-curve, two cut-off points (29th and 94th percentile) predicting opposite features were defined. In contrast to *SMARCA4* high *EZH2* mRNA levels proved to be associated with impaired PFS (*P* = 0.025) in the entire OC collective when the threshold for dichotomization between high and low was set at the 29th percentile. However, for OS no clinical prognostic impact was delineated in univariate survival analysis at this threshold (Fig. 3). Moreover, in multivariate analysis the prognostic impact of *EZH2* transcript levels could be confirmed for PFS in the whole collective of OCs (HR 1.62, *P* = 0.034) (Table 2) and in the subgroup of “high-grade OCs” (HR 1.67, *P* = 0.045) (Table 3). No independent impact of *EZH2* mRNA levels on clinical outcome was revealed when the subgroup of “low-grade OCs” was analyzed separately for PFS and OS.

**Figure 4:**
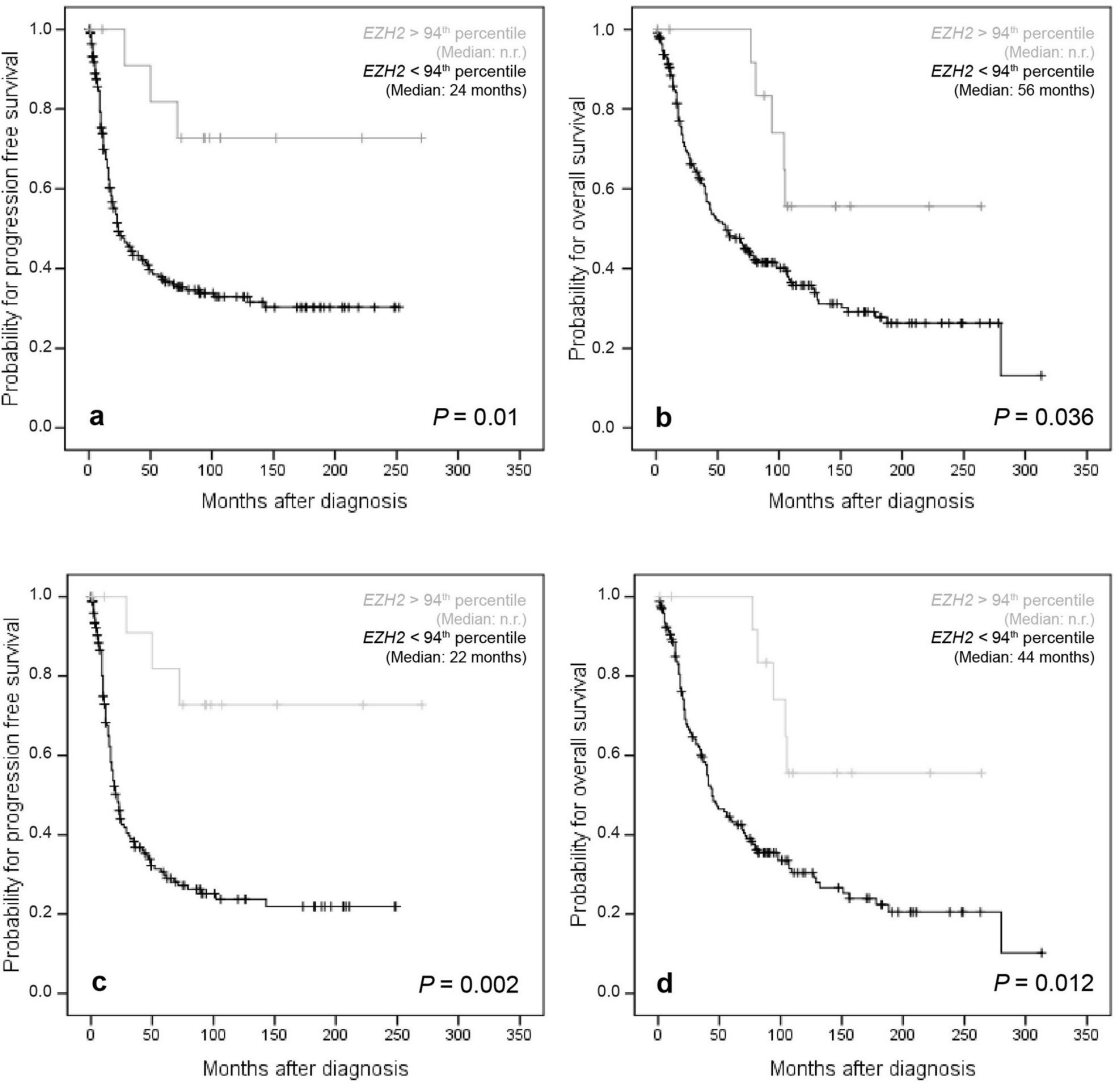
Kaplan–Meier survival analysis and *EZH2* mRNA expression according to the 94th percentile as cut off-value in the whole cohort and in the subgroup of “high-grade OC” patients. (**a**) Progression free survival in 238 OC patients, (**b**) overall survival in 238 OC patients, (**c**) progression free survival in 186 “high-grade OC” patients, (**d**) overall survival in 186 “high-grade OC” patients; *n.r.* not reached.

However, when the 94th percentile was used for cohort dichotomization, a conversion of the prognostic attribute of *EZH2* expression from unfavorable to favorable clinical outcome became apparent for both the PFS and OS. Thus, with these percentiles very high *EZH2* mRNA levels were associated with improved PFS (*P* = 0.01) and OS (*P* = 0.036) in the whole investigated cohort and in the subgroup of “high-grade OCs” (*P* = 0.002 and *P* = 0.012, respectively) (Fig. 4). It is worth noting that all tumors classified as ultra-high *EZH2* expressing were all high-grade cancers. Moreover, Cox-regression analysis confirmed the independency of prognostic relevance of ultra-high *EZH2* mRNA expression for PFS and OS in the whole cohort (HR 0.21, *P* = 0.009 and HR 0.4, *P* = 0.032, respectively) (Table 2) and for PFS in the subgroup of “high-grade OCs” (HR 0.2, *P* = 0.007) (Table 3).

### Validation of *SMARCA4* and *EZH2* survival analysis in independent cohorts

Survival analyses employing mRNA expression data from the Niigata OC cohort (n = 110) and the MSKCC OC cohort (n = 185) confirmed the prognostic relevance of *SMARCA4*. High *SMARCA4* mRNA expression was associated with a better survival (Niigata cohort: PFS: *P* = 0.011, OS: *P* = 0.014; MSKCC cohort: PFS: *P* = 0.003; OS: *P* = 0.009; Supplementary Fig. S1).

Prognostic relevance was also confirmed for EZH2, where high *EZH2* mRNA expression was associated with poor PFS in the Niigata cohort (cut-off: 12th percentile; *P* = 0.048), which in contrast to our cohort was translated in impaired OS (cut-off: 12th percentile; *P* = 0.015). The latter finding was corroborated by the Tothill cohort (cut-off: 25th percentile *P* = 0.020) (Supplementary Fig. S2).

Extremely high cut-offs used for cohort dichotomization revealed no conversion of the prognostic attribute of *EZH2* expression as observed in our data. Interestingly, however, in the Duke cohort high *EZH2* mRNA expression was associated with an improved OS (*P* = 0.008) when a high *EZH2* expression cut-off (69th percentile) was used (Supplementary Fig. S2).

## Discussion

In the present work expression of *SMARCA4* and *EZH2* as two key players of the functionally antagonist DNA remodeling complexes SWI/SNF and PRC2, respectively, were analyzed on the transcriptome level in OCs. Whereas the SWI/SNF complex opens accessibility to chromatin, especially to tumor-suppressor genes, by removal of PCR2 and promotes cellular differentiation, the PRC2 complex, in contrast, stalls differentiation and favors malignant transformation. Imbalances between both complexes can be due to overexpression of *EZH2* in undifferentiated cancers, but most commonly occur in cancers by mutations of members of the SWI/SNF complex, such as *ARID1A* and *SMARCA4* that together with *SMARCA2* is the ATPase subunit of this tumor-suppressing complex. Such imbalances lead to a supremacy of *EZH2*, the catalytic subunit of PRC2, which is the exclusive human methyltransferase for H3K27. Aberrant tri-methylated H3K27 represents a transcriptionally repressive histone mark that is highly oncogenic in a broad spectrum of human cancers.

This dualistic behavior regarding both complexes is well reflected in the herein performed expression analyses of *SMARCA4* and *EZH2* in terms that high expression of *SMARCA4* independently reflects a favorable PFS and OS whereas elevated EZH2 was independently associated with improved clinical outcome. Moreover, high *SMARCA4* mRNA levels are associated with better surgical resectability, especially in high-grade cancers. This is of importance, since the residual disease after primary debulking surgery is the most important prognostic factor in OC and is often wrongly attributed to surgical skills only. Albeit associated with cell-cycle promoting *E2F3a*, a known prognosticator in OC^31^, high *SMARCA4* expressing cancers were associated with improved survival. However, Guerrero-Martinez and Reyes showed high expression of *SMARCA4* to be associated with worse prognosis in OC^32^. These findings are not only in conflict with our data but also with those of the Niigata OC cohort and the MSKCC cohort.

Our data show that high *EZH2* transcript levels were related to high-grade, *BRCA1*-mutated ovarian cancers, which in turn were very strongly associated with cell cycle promoting factors *E2F1* and *E2F3a*. All this indicates that *EZH2* high expressing cancers exhibit a very high proliferative turnover. A fact that is additionally underscored by the significant relationship with the expression of *BRCA1* and -2 mRNA, which is thought to reflect high DNA repair events known to occur at higher rates in tumors with high mitotic activity^33^. Thus, it is not unexpected that overexpression of growth promoting and oncogenic *EZH2* is associated with impaired PFS in univariate and multivariate survival analysis. Accordingly, elevated *EZH2* expression was also associated with adverse prognosis in several other tumor entities, such as breast, cervical, larynx and colorectal cancers^34–37^. Rao et al. also showed that high *EZH2* expression was associated with poor prognosis in OC and observed a positive correlation between overexpression of *EZH2* and TGF-β1 suggesting a potential role of *EZH2* in the control of cell migration and invasion via regulation of TGF-β1 expression^38^. A further hint for *EZH2* promoting effects on migration and invasion was provided by Yi et al. who showed that *EZH2* inhibits the expression of the physiological matrix metalloproteinase inhibitor TIMP2 in ovarian cancer cells^39^. Moreover, in OC *EZH2* upregulation via inhibition of its negative regulator miRNA-137 by c-Myc has been implicated into promotion of cisplatin resistance^40^.

However, due to the S-shaped ROC curve in the calculation of the Youden’s Index we herein uncovered that at very high *EZH2* transcript levels the effect on survival converted from unfavorable to favorable PFS and OS. In contrast to our findings of poor prognosis at lower threshold expression, outcome improvements were not only observed for PFS but also for OS and were furthermore statistically confirmed in the multivariate Cox-regression analysis. We hypothesize that this conversion is not due simply to statistical unevenness but may be the result of different biological effects, which occur after a critical *EZH2* activity is reached in the concerned tumor cells. Very recently, the inhibitory role of *EZH2* on the activity of *MAD2L2* (Rev7), an essential subunit of the shieldin complex, was highlighted. Shieldin/Rev7 protects DNA broken ends from a 5′-3′ resection at double strand breaks (DSB), which is essential that *RAD51*-dependent homologous recombination (HR) repair can occur^29^. Thus, ultra-high *EZH2* activity favors HR repair over non-homologous end-joining (NHEJ), by lowering Rev7 activity. In line with this, Xu et al.^41^ reported that mutation of Rev7 or abrogation of its activity was found to increase HR capacity and to confer resistance to PARP inhibitors on the one hand but hypersensitivity toward platinum containing drugs on the other hand^41–43^. As almost all of the herein included patients were treated with platinum-based chemotherapy but none of them were treated with a PARP inhibitor in any of the lines of treatment, it is conceivable that the observed survival advantage in the small percentage of patients with ultra-high *EZH2* expressing tumors may originate from improved responsiveness to platinum-based treatment. Detailed analysis of the *EZH2* ultra-high cohort revealed that all these cancers in fact, exhibited platinum-hypersensitivity and were without exception advanced high-grade tumors. For comparison, in the stage-adjusted cohort of high-grade cancers with lower *EZH2* expression, the rate of platinum-hypersensitivity was as low as 17.5% (data not shown). In accordance, loss of *EZH2* has been recently described to drive resistance to carboplatin and paclitaxel in serous OCs provided *ATM* is upregulated^44^. These findings should give cause for concern regarding an indiscriminative use of *EZH2* inhibitors combined with platinum-based chemotherapy in OC patients.

*EZH2* is a good example showing that a biomarker can change its prognostic attributes with the levels of its expression in the same tumor entity. Such conversions may be dependent on a specific molecular context in the concerned cancers, for instance regarding *EZH2* this could be either a concomitant overexpression of *ATM* or changing features in the interplay with Rev7^29,44^.

## Methods

### Patients and samples

Ovarian tissue samples from 238 patients with epithelial OCs were obtained at primary debulking surgery in the period from 2006 to 2017. Patients were 22–90 years old and the median age at the time of diagnosis was 62 years. Additionally, nineteen non-neoplastic fallopian tubal tissue specimens and 16 non-neoplastic ovarian tissues were used as a control group. These samples were obtained from elective salpingo-oophorectomies for benign conditions (i.e. salpingectomy for sterilization or contralateral salpingo-oophorectomy as part of surgery for benign cysts). Median age at time of surgery was 52 years (range 19–81 years). The tissue probes were collected and processed at the Pathology Unit of the Department of Obstetrics and Gynecology of the Medical University of Innsbruck, Austria.

Written informed consent was obtained from all patients before enrolment. The study was reviewed and approved by the Ethics committee of the Medical University of Innsbruck (reference number: 1157/2018) and conducted in accordance with the Declaration of Helsinki. All samples were anonymized before the analysis. The median follow-up period was 20 months (IQR 9–73 months) regarding the progression-free survival and 45 months (IQR 18–107 months) concerning overall survival. Clinico-pathological characteristics of included patients are listed in Table 1.

Analyses were performed in the whole OC cohort as well as in two subgroups— “high-grade OCs” and “low-grade OCs”, separately. For the latter evaluations, the final subdivision was made in accordance with the WHO classification of 2020 by a second pathologic review^45^. In these subgroup analyses mucinous cancers were excluded.

### Quantitative real time PCR

Total cellular RNA extraction from and reverse transcription were performed as previously described^33^.

Primers and probes for the TATA box-binding protein (TBP; a component of the DNA-binding protein complex TFIID as an endogenous RNA control) were used according to Bieche et al.^46^. Primers and probes for *EZH2* and *SMARCA4* were purchased from Applied Biosystems (Foster City, CA, USA, Applied Biosystems Assay ID: Hs00544830_m1 and Hs00231324_m1). PCR reactions were performed as previously described^33^. The standard curves were generated using serially diluted solutions of standard cDNA derived from the HTB-77 carcinoma cell line. Each experiment included this standard curve, a positive control sample (OVCAR3 carcinoma cell-line for *EZH2*; HOC7 for *SMARCA4*), 40 patient samples and a no template control. Real-time PCR assays were conducted in duplicates for each sample, and the mean value was used for calculation. The expression analyses of *BRCA1*, *BRCA2*, *E2F1* and *E2F3a* were recently described^31,33^.

Data normalization was carried out against TBP expression, the endogenous RNA-control and expressed in arbitrary units.

### Mutation analysis

Genomic DNA from pulverized, quick-frozen OC specimens was isolated using the DNeasy tissue-kit (Qiagen, Hilden, Germany). Targeted NGS was performed using the TruSight Cancer sequencing panel (Illumina, San Diego, USA) as described recently^33^.

### Validation cohort

To validate findings from our study cohort, we employed gene expression data from independent cohorts of OC patients publically available at GEO, a public functional genomics data repository (GSE17260 (Niigata, Yoshihara cohort), GSE9891 (AOCS, RBH, WH, NKI-AVL, Tothill cohort), GSE26712 (MSKCC, Bonome cohort)) and available via PrognoScan, a new database for meta-analysis of the prognostic value of genes (DUKE-OC, Bild cohort)^47^.

### Statistical analysis

The non-parametric Mann–Whitney *U* test or Kruskal–Wallis test were applied to test for statistical significance between two groups or more than two groups, respectively. To assess the correlations between *SMARCA4* mRNA-expression*, EZH2* mRNA-expression and different other molecular markers Spearman-rank correlation analyses were used.

Progression-free survival (PFS) was defined as the time from diagnosis of the primary tumor to the histopathological confirmation of recurrence. Overall survival (OS) was defined as the time from diagnosis to death from any cause or to the last clinical inspection. Univariate Kaplan–Meier analyses and multivariate Cox-regression survival analyses were used to explore the association of *SMARCA4* mRNA expression and *EZH2* mRNA expression with PFS and OS.

In order to investigate the biological impact of *SMARCA4* and *EZH2* expression on the basis of the clinical outcome in the cohort, Youden’s Index^48^ was used to identify the optimal threshold for both parameters to distinguish between “low” and “high” expression. The optimal discriminatory cut-off point for *SMARCA4* corresponded to the 30th percentile. As for *EZH2* the Receiver Operating Characteristics (ROC) curve was S-shaped and crossed the diagonal random classifier line, which separates both areas of opposite characteristics, two discriminatory cut-off points were defined for *EZH2*: the one at the 29th and the second at the 94th percentile.

*P *values < 0.05 were considered as statistically significant. Statistical analysis was performed using SPSS statistical software (version 20.0.0; SPSS Inc., Chicago, IL, USA).

### Ethics approval

The study was reviewed and approved by the Ethics committee of the Medical University of Innsbruck (reference number: 1157/2018) and conducted in accordance with the Declaration of Helsinki.

### Consent for publication

Written informed consent was obtained from all patients before enrolment.

## Supplementary information

Supplementary Tables and Legends.

Supplementary Table S4.

Supplementary Figures.

## Data Availability

The source dataset is shown in Supplementary Table S4.
