# Supplementary material for: Clinical impact of EZH2 and its antagonist SMARCA4 in ovarian cancer
Source: Sci Rep. 2020 Nov 23;10:20412. doi: 10.1038/s41598-020-77532-x (PMC7684284; doi:10.1038/s41598-020-77532-x)
Supplement: Supplementary file 1 — Supplementary Tables and Legends. [file 41598_2020_77532_MOESM1_ESM.docx]

**Supplementary Material – Tables**

**Clinical impact of *EZH2* and its antagonist *SMARCA4* in ovarian cancer**

Katharina Leitner, Irina Tsibulak, Verena Wieser, Katharina Knoll, Daniel Reimer, Christian Marth, Heidi Fiegl and Alain G. Zeimet^*^

Department of Obstetrics and Gynecology, Innsbruck Medical University, Innsbruck, Austria

^*^ **Correspondence and reprint requests:**

Alain G. Zeimet M.D.

Department of Obstetrics and Gynecology

Innsbruck Medical University

Anichstraße 35

6020 Innsbruck, Austria

Tel +43 512 504-23051

Fax +43 512 504-23055

E-mail: alain.zeimet@i-med.ac.at

**Supplementary Table S1: Association of *SMARCA4* mRNA expression with clinico-pathological characteristics in 25 “low-grade OCs” and 186 “high-grade OCs”.**

|  | | ***SMARCA4* mRNA expression values** (arbitrary units) | | | | | |
| --- | --- | --- | --- | --- | --- | --- | --- |
|  | | ***“low-grade OC”*** | | | ***“high-grade OC”*** | | |
| Variable | | Number  (%) | Median  (IQR) | *P* value | Number (%) | Median  (IQR) | *P* value |
| ***Age*** | |  |  |  |  |  |  |
| ≤ 62 years (median age) | | 14 (56.0 %) | 1.963 (1.62-2.30) | 0.893 | 93 (50.0 %) | 2.437  (1.79-3.49) | 0.051 |
| > 62 years | | 11  (44.0 %) | 1.885 (1.56-2.66) |  | 93 (50.0 %) | 2.076  (1.37-2.93) |  |
| ≤ 51.5 years | | 10 (40.0 %) | 1.961 (1.54-2.16) | 0.643 | 35 (18.8 %) | 2.538  (1.82-4.01) | 0.094 |
| > 51.5 years | | 15  (60.0 %) | 1.917  (1.65-2.66) |  | 151 (81.2 %) | 2.099  (1.61-2.89) |  |
| *≤ 70 years* | | *19*  *(76.0 %)* | *2.009 (1.84-2.44)* | *0.366* | *131 (70.4 %)* | *2.400*  *(1.76-3.44)* | ***0.027*** |
| *> 70 years* | | *6*  *(24.0 %)* | *1.659*  *(1.48-2.70)* |  | *55*  *(29.6 %)* | *2.005*  *(1.17-2.86)* |  |
| *≤ 40 years* | | *4*  *(16.0 %)* | *1.961*  *(1.63-2.16)* | *0.858* | *8*  *(4.3 %)* | *2.222*  *(1.07-2.88)* | *0.564* |
| *> 40 years* | | *21*  *(84.0 %)* | *1.917 (1.61-2.61)* |  | *178*  *(95.7 %)* | *2.250*  *(1.65-3.10)* |  |
| ***FIGO stage*** | |  |  |  |  |  |  |
| I/II | | 15  (60.0 %) | 2.093  (1.65-2.66) | 0.397 | 39  (21.0 %) | 2.751  (1.94-3.78) | 0.058 |
| III/IV | | 10  (40.0 %) | 1.876  (1.52-2.30) |  | 147  (79.0 %) | 2.089  (1.62-2.92) |  |
| ***Residual disease*** | |  |  |  |  |  |  |
| Complete debulking | | 22  (88.0 %) | 1.963  (1.56-2.59) | 0.969 | 77  (41.4 %) | 2.467  (1.83-3.72) | **0.016** |
| Any tumor residual | | 3  (12.0 %) | 1.914  (1.84-.) |  | 100  (53.8 %) | 2.079  (1.44-2.81) |  |
| Not indicated | | - | - |  | 9  (4.8 %) | - |  |
| ***BRCA1 mutation status*** | |  |  |  |  |  |  |
| Wild-type | | 19  (76.0 %) | 1.917  (1.56-2.56) | 0.785 | 133  (71.5 %) | 2.216  (1.66-3.02) | 0.764 |
| *BRCA1* mutated | | 4  (16.0 %) | 1.975  (1.70-3.81) |  | 31  (16.7 %) | 2.447  (1.79-3.20) |  |
| Not indicated | | 2  (8.0 %) | - |  | 22 (11.8 %) |  |  |
| ***BRCA2 mutation status*** | |  |  |  |  |  |  |
| Wild-type | | 23 (92.0 %) | 1.917 (1.92-2.56) |  | 155  (83.3 %) | 2.228  (1.68-3.07) | 0.726 |
| *BRCA2* mutated | | - |  |  | 9  (4.8 %) | 1.904  (1.55-3.37) |  |
| Not indicated | | 2 (8.0 %) |  |  | 22 (11.8 %) | - |  |
|  | |  |  |  |  |  |  |
| QR – Interquartile range.  Bold values indicate *P* < 0.05. | | | | | | | |

**Supplementary Table S2: Association of *EZH2* mRNA expression with clinico-pathological characteristics in 25 “low-grade OCs” and 186 “high-grade OCs”.**

|  | | ***EZH2* mRNA expression values (arbitrary units)** | | | | | |
| --- | --- | --- | --- | --- | --- | --- | --- |
|  | | ***“low-grade OCs”*** | | | ***“high-grade OCs”*** | | |
| Variable | | Number (%) | Median  (IQR) | *P* value | Number (%) | Median  (IQR) | *P* value |
| ***Age*** | |  |  |  |  |  |  |
| ≤ 62 years (median age) | | 14 (56.0 %) | 1.225 (0.68-2.03) | 0.107 | 93 (50.0 %) | 2.620  (1.64-4.34) | 0.136 |
| > 62 years | | 11  (44.0 %) | 2.520  (1.25-3.20) |  | 93 (50.0 %) | 2.230  (1.42-3.24) |  |
| ≤ 51.5 years | | 10 (40.0 %) | 0.785  (0.63-1.70) | **0.031** | 35 (18.8 %) | 2.270  (1.22-4.28) | 0.837 |
| > 51.5 years | | 15  (60.0 %) | 2.300  (1.30-3.11) |  | 151 (81.2 %) | 2.420  (1.52-3.47) |  |
| *≤ 70 years* | | *19*  *(76.0 %)* | *1.590 (0.73-2.61)* | *1.000* | *131*  *(70.4 %)* | *2.450*  *(1.60-4.25)* | *0.138* |
| *> 70 years* | | *6*  *(24.0 %)* | *1.900*  *(0.60-2.78)* |  | *55*  *(29.6 %)* | *2.140*  *(1.25-3.22)* |  |
| *≤ 40 years* | | *4*  *(16.0 %)* | *0.940*  *(0.68-1.48)* | *0.177* | *8*  *(4.3 %)* | *1.500*  *(0.69-3.73)* | *0.119* |
| *> 40 years* | | *21*  *(84.0 %)* | *2.030*  *(0.76-2.86)* |  | *178*  *(95.7 %)* | *2.435*  *(1.53-3.87)* |  |
| ***Residual disease*** | |  |  |  |  |  |  |
| Complete debulking | | 22  (88.0 %) | 1.590 (0.71-2.74) | 0.723 | 77  (41.4 %) | 2.420  (1.51-4.32) | 0.614 |
| Any tumor residual | | 3  (12.0 %) | 1.590  (0.69-.) |  | 100  (53.8 %) | 2.390  (1.49-3.81) |  |
| Not indicated | | - | - |  | 9  (4.8 %) | - |  |
| ***BRCA1 mutation status*** | |  |  |  |  |  |  |
| Wild-type | | 19  (76.0 %) | 1.590  (0.66-2.61) | 0.611 | 133  (71.5 %) | 2.190  (1.41-3.41) | **0.013** |
| *BRCA1* mutated | | 4  (16.0 %) | 1.975  (0.73-3.43) |  | 31  (16.7 %) | 3.070  (1.93-4.71) |  |
| Not indicated | | 2  (8.0 %) | - |  | 22 (11.8 %) |  |  |
| ***BRCA2 mutation status*** | |  |  |  |  |  |  |
| Wild-type | | 23 (92.0 %) | 1.590 (0.69-2.61) | - | 155  (83.3 %) | 2.370  (1.50-3.71) | 0.421 |
| *BRCA2* mutated | | - | - |  | 9  (4.8 %) | 3.910  (1.10-7.05) |  |
| Not indicated | | 2 (8.0 %) | - |  | 22 (11.8 %) | - |  |
| IQR – Interquartile range.  Bold values indicate *P* < 0.05. | | | | | | | |

**Supplementary Table S3: Correlations between *SMARCA4* mRNA, *EZH2* mRNA expression and transcriptomic expression of other molecular markers.**

| Variable  (mRNA expression) | Number | ***SMARCA4* mRNA expression** | | ***EZH2* mRNA expression** | |  |
| --- | --- | --- | --- | --- | --- | --- |
|  |  | Correlation coefficient (r^s^) | *P* value | Correlation coefficient (r^s^) | *P* value |  |
| **A Correlation analysis in 238 ovarian cancer patients** | | | | | |  |
| *SMARCA4* | 238 | 1.000 | - | 0.392 | **< 0.001** |  |
| *EZH2* | 238 | 0.392 | **< 0.001** | 1.000 | - |  |
| *BRCA1* | 190 | 0.229 | **0.002** | 0.293 | **< 0.001** |  |
| *BRCA2* | 166 | 0.264 | **0.001** | 0.679 | **< 0.001** |  |
| *E2F1* | 34 | 0.163 | 0.357 | 0.788 | **< 0.001** |  |
| *E2F3a* | 77 | 0.394 | **< 0.001** | 0.684 | **< 0.001** |  |
| **B Correlation analysis in 186 “high-grade” ovarian cancer patients** | | | | | |  |
| *SMARCA4* | 186 | 1.000 | - | 0.384 | **< 0.001** |  |
| *EZH2* | 186 | 0.384 | **< 0.001** | 1.000 | - |  |
| *BRCA1* | 164 | 0.206 | **0.008** | 0.276 | **< 0.001** |  |
| *BRCA2* | 142 | 0.236 | **0.005** | 0.677 | **< 0.001** |  |
| *E2F1* | 30 | 0.149 | 0.432 | 0.754 | **< 0.001** |  |
| *E2F3a* | 70 | 0.392 | **0.001** | 0.675 | **< 0.001** |  |
| To assess the correlations Spearman-rank correlation analyses were used.  r^s^ – Correlation coefficient. Bold values indicate *P* < 0.05. | | | | | | |

**Supplementary Table S4: Dataset with clinico-pathological characteristics and *SMARCA4* and *EZH2* expression values.**

Excel File “Supplementary Material Table S4”
