# Supplementary material for: Clinical impact of EZH2 and its antagonist SMARCA4 in ovarian cancer
Source: Sci Rep. 2020 Nov 23;10:20412. doi: 10.1038/s41598-020-77532-x (PMC7684284; doi:10.1038/s41598-020-77532-x)
Supplement: Supplementary file 3 — Supplementary Figures. [file 41598_2020_77532_MOESM3_ESM.docx]

**Supplementary Material – Figures**

**Clinical impact of *EZH2* and its antagonist *SMARCA4* in ovarian cancer**

Katharina Leitner, Irina Tsibulak, Verena Wieser, Katharina Knoll, Daniel Reimer, Christian Marth, Heidi Fiegl and Alain G. Zeimet^*^

Department of Obstetrics and Gynecology, Innsbruck Medical University, Innsbruck, Austria

^*^ **Correspondence and reprint requests:**

Alain G. Zeimet M.D.

Department of Obstetrics and Gynecology

Innsbruck Medical University

Anichstraße 35

6020 Innsbruck, Austria

Tel +43 512 504-23051

Fax +43 512 504-23055

E-mail: alain.zeimet@i-med.ac.at

**Supplementary Fig. S1:**

**Kaplan-Meier survival analysis and *SMARCA4* mRNA expression in independent cohorts.**

**
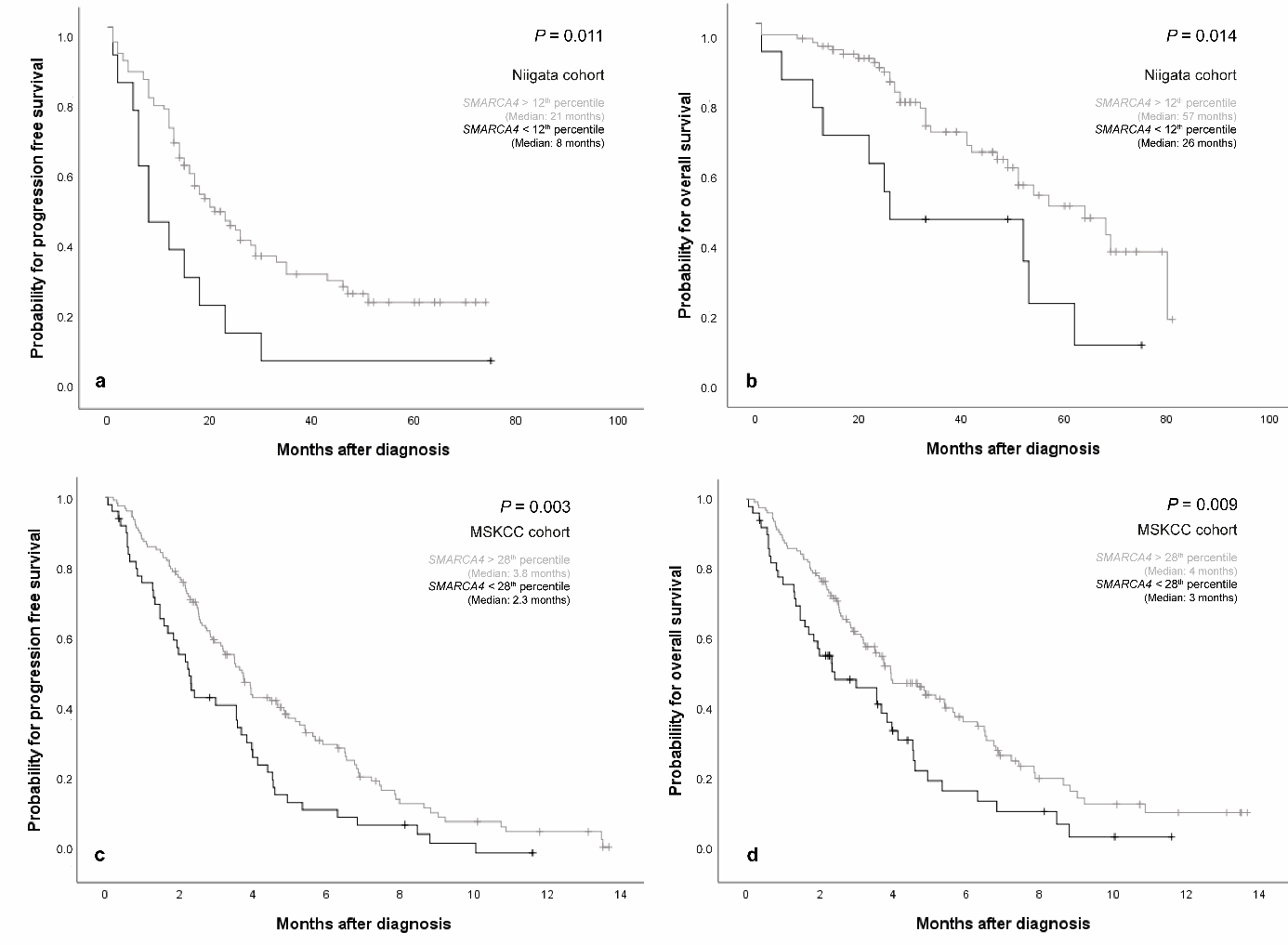
**

(a) Progression free survival and (b) overall survival in the GSE17260, Niigata cohort,
and (c) progression free and (d) overall survival in the GSE26712, MSKCC cohort.

**Supplementary Fig. S2:**

**Kaplan-Meier survival analysis and *EZH2* mRNA expression in independent cohorts.**

**
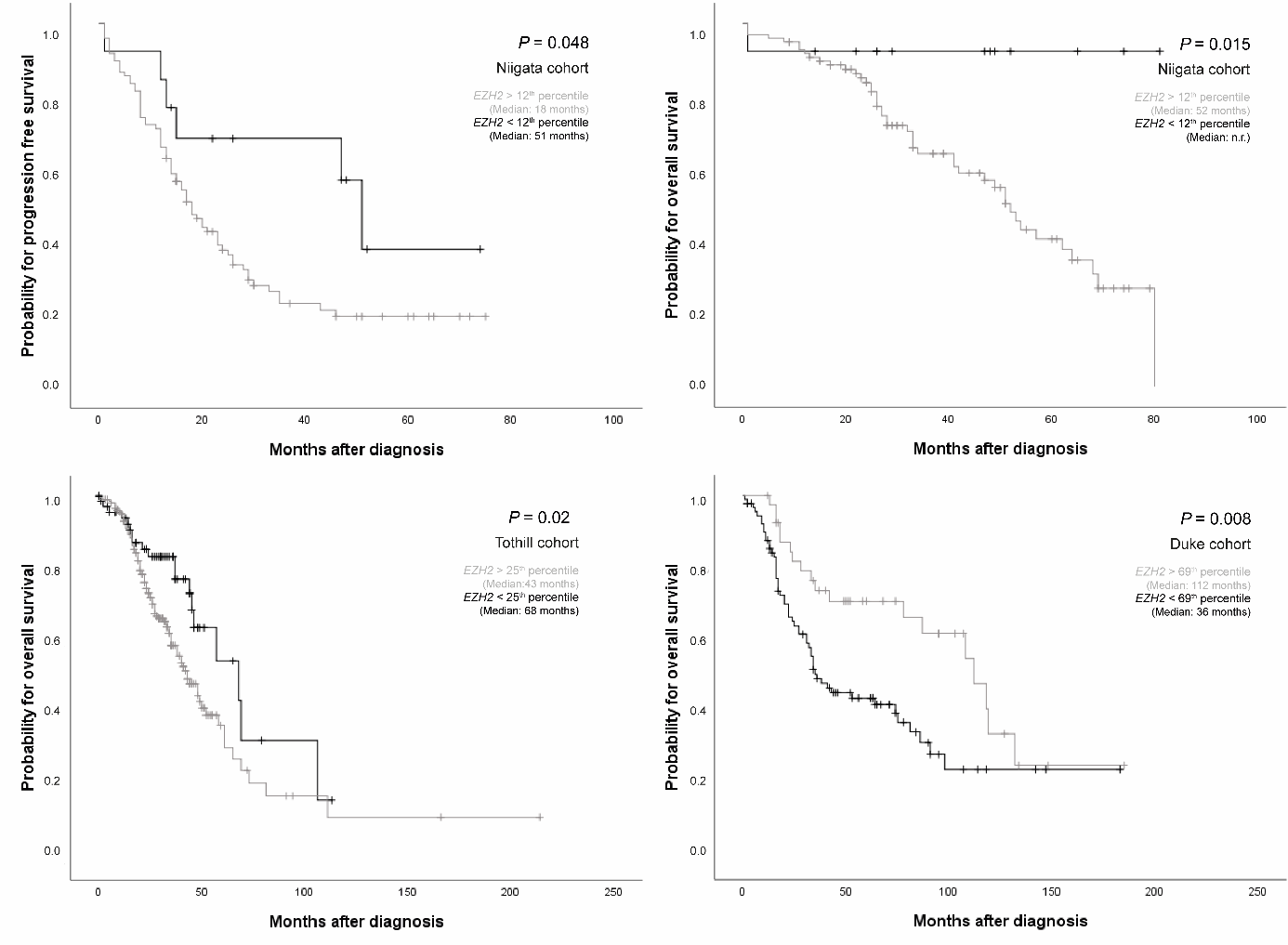
**

(a) Progression free survival and (b) overall survival in the GSE17260, Niigata cohort,
(c) overall survival in the GSE9891, Tothill cohort and (d) overall survival in the DUKE-OC cohort.
